# 2,2′-(Heptane-1,7-di­yl)dibenz­imidazo­lium chloride nitrate monohydrate

**DOI:** 10.1107/S1600536809006370

**Published:** 2009-02-28

**Authors:** Jin-Ping Zeng, Yun-Qian Zhang, Sai-Feng Xue, Qian-Jiang Zhu, Zhu Tao

**Affiliations:** a Key Laboratory of Macrocyclic and Supramolecular Chemistry of Guizhou Province, Guizhou University, Guiyang 550025, People’s Republic of China; b Institute of Applied Chemistry, Guizhou University, Guiyang 550025, People’s Republic of China

## Abstract

In the title compound, C_21_H_26_N_4_^2+^·Cl^−^·NO_3_^−^·H_2_O, the organic cations, anions and water mol­ecules are linked through N—H⋯Cl, N—H⋯O, N—H⋯N and O—H⋯Cl hydrogen bonds, forming a three-dimensional framework, assisted by C—H⋯π inter­actions.

## Related literature

For general background regarding inter­actions of linear polyaromatic compounds with cucurbit[*n*]urils, see: Day & Arnold (2000 ▶); Day *et al.* (2002 ▶); Freeman *et al.* (1981 ▶); Kim *et al.* (2000 ▶). For the synthesis, see: Wang & Joullié (1957 ▶).
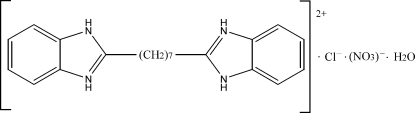


## Experimental

### 

#### Crystal data

C_21_H_26_N_4_^2+^·Cl^−^·NO_3_^−^·H_2_O
*M* *_r_* = 449.93
Orthorhombic, 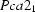

*a* = 24.462 (10) Å
*b* = 5.102 (2) Å
*c* = 18.210 (7) Å
*V* = 2272.5 (15) Å^3^
*Z* = 4
Mo *K*α radiation
μ = 0.21 mm^−1^
*T* = 293 K
0.31 × 0.22 × 0.18 mm

#### Data collection

Bruker SMART APEXII CCD area-detector diffractometer
Absorption correction: multi-scan (*SADABS*; Bruker, 2005 ▶) *T*_min_ = 0.939, *T*_max_ = 0.964
14275 measured reflections
2086 independent reflections
1619 reflections with *I* > 2σ(*I*)
*R*_int_ = 0.063

#### Refinement

*R*[*F* ^2^ > 2σ(*F* ^2^)] = 0.038
*wR*(*F* ^2^) = 0.084
*S* = 1.03
2086 reflections
288 parameters
5 restraints
H atoms treated by a mixture of independent and constrained refinement
Δρ_max_ = 0.19 e Å^−3^
Δρ_min_ = −0.17 e Å^−3^

### 

Data collection: *APEX2* (Bruker, 2005 ▶); cell refinement: *SAINT* (Bruker, 2005 ▶); data reduction: *SAINT*; program(s) used to solve structure: *SHELXS97* (Sheldrick, 2008 ▶); program(s) used to refine structure: *SHELXL97* (Sheldrick, 2008 ▶); molecular graphics: *ORTEP-3 for Windows* (Farrugia, 1997 ▶); software used to prepare material for publication: *WinGX* (Farrugia, 1999 ▶).

## Supplementary Material

Crystal structure: contains datablocks global, I. DOI: 10.1107/S1600536809006370/ez2159sup1.cif

Structure factors: contains datablocks I. DOI: 10.1107/S1600536809006370/ez2159Isup2.hkl

Additional supplementary materials:  crystallographic information; 3D view; checkCIF report

## Figures and Tables

**Table 1:** Hydrogen-bond geometry (Å, °)

*D*—H⋯*A*	*D*—H	H⋯*A*	*D*⋯*A*	*D*—H⋯*A*
N1—H1*A*⋯Cl1	0.86	2.22	3.066 (3)	168
N2—H2*A*⋯O3^i^	0.86	1.94	2.778 (4)	164
N2—H2*A*⋯O1^i^	0.86	2.53	3.243 (4)	141
N2—H2*A*⋯N5^i^	0.86	2.59	3.436 (5)	168
N3—H3*A*⋯O2^ii^	0.86	1.95	2.807 (4)	177
N4—H4*A*⋯O1*W*^iii^	0.86	1.87	2.730 (4)	173
O1*W*—H1*WA*⋯Cl1	0.927 (19)	2.19 (2)	3.088 (4)	163 (4)
O1*W*—H1*WB*⋯Cl1^iv^	0.83 (2)	2.31 (3)	3.099 (3)	159 (5)
C10—H10*A*⋯*Cg*3^v^	0.97	3.36	4.148 (4)	140
C11—H11*B*⋯*Cg*4^vi^	0.97	3.21	4.047 (4)	146

Symmetry codes: (i) 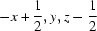; (ii) 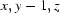; (iii) 
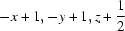
; (iv) 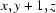; (v) 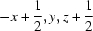; (vi) 
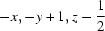
. *Cg*3 and *Cg*4 are the centroids of the C1–C6 and C16–C21 benzene rings, respectively.

**Fig. 1.**
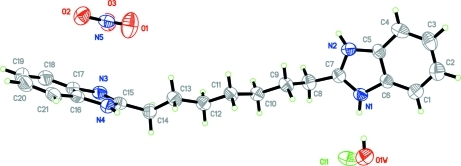
The molecular structure of (I), shown with the atom-labelling scheme and 50% probability displacement ellipsoids.

## supplementary crystallographic information

### Comment

This paper describes the preparation and structure of a new linear polyaromatic compound (I) in which the multiple functional groups can develop strong intermolecular interactions with cucurbit[*n*]urils (CB[*n*]) (Freeman *et al.*,1981; Day & Arnold, 2000; Day *et al.*, 2002; Kim *et al.*, 2000).

The molecular structure of (I), shown in Fig. 1, consists of one organic cation, one Cl^-^ anion, one NO_3_^-^ anion and one lattice water molecule. The two benzimidazole groups of the organic cation are not co-planar, but are oriented at a dihedral angle of 78.42 (6) ° with respect to each other. Molecules are linked *via* an N1—H1A···Cl1, N2—H2A···O3, N2—H2A···O1, N2—H2A···N5, N3—H3A···O2, N4—H4A···O1W and O1W—H1WA···Cl1 network of hydrogen bonds (Table 1) forming a three-dimensional framework. In addition, C—H···π interactions occur between adjacent organic cations (Table 1, *Cg*(3) and *Cg*(4) are the centroids of the C1—C6 and C16—C21 benzene rings, respectively).

### Experimental

A solution of *o*-phenylenediamine (5.40 g, 0.05 mol) and azelaic acid (4.71 g, 0.025 mol) were refluxed for twelve hours in 50 ml of 4*M* HCl. The reaction mixture was then cooled for one day and the blue crystalline 2,2'-(Heptane-1,7-diyl)dibenzimidazolium dihydrochloride which separated was removed by filtration and dried (Wang *et al.*, 1957). Yield: 31%. The dihydrochloride (2.03 g, 5 mmol) and lanthanum nitrate (3.25 g, 10 mmol) were refluxed for three h in 50 ml water, and the mixture was cooled and filtered. Upon standing at room temperature, crystals of title compound (I) were obtained after several days.

### Refinement

The water H atoms were located in a difference Fourier synthesis and refined with distances restrained to O—H = O.82 (2) Å and H—H =1.37 (4) Å, with *U*_iso_(H) =1.2*U*_eq_(O). All other H atoms were placed in calculated positions and refined as riding, with C—H = 0.93–0.97 Å, N—H = 0.86 Å, and with *U*_iso_(H) = 1.2 *U*_eq_ (C, N). In the absence of significant anomalous scattering, Friedel equivalents (900 pairs) were merged before the final refinement.

### Crystal data

**Table:**

C_21_H_26_N_4_^2+^·Cl^−^·NO_3_^−^·H_2_O	*F*(000) = 952
*M**_r_* = 449.93	*D*_x_ = 1.315 Mg m^−^^3^
Orthorhombic, *P**c**a*2_1_	Mo *K*α radiation, λ = 0.71073 Å
Hall symbol: P 2c -2ac	Cell parameters from 3986 reflections
*a* = 24.462 (10) Å	θ = 1.7–25.1°
*b* = 5.102 (2) Å	µ = 0.21 mm^−^^1^
*c* = 18.210 (7) Å	*T* = 293 K
*V* = 2272.5 (15) Å^3^	Prism, colorless
*Z* = 4	0.31 × 0.22 × 0.18 mm

### Data collection

**Table:**

Bruker SMART APEXII CCD area-detector diffractometer	2086 independent reflections
Radiation source: fine-focus sealed tube	1619 reflections with *I* > 2σ(*I*)
graphite	*R*_int_ = 0.063
φ and ω scans	θ_max_ = 25.1°, θ_min_ = 1.7°
Absorption correction: multi-scan (*SADABS*; Bruker, 2005)	*h* = −26→29
*T*_min_ = 0.939, *T*_max_ = 0.964	*k* = −6→6
14275 measured reflections	*l* = −21→21

### Refinement

**Table:**

Refinement on *F*^2^	Primary atom site location: structure-invariant direct methods
Least-squares matrix: full	Secondary atom site location: difference Fourier map
*R*[*F*^2^ > 2σ(*F*^2^)] = 0.038	Hydrogen site location: inferred from neighbouring sites
*wR*(*F*^2^) = 0.084	H atoms treated by a mixture of independent and constrained refinement
*S* = 1.03	*w* = 1/[σ^2^(*F*_o_^2^) + (0.0385*P*)^2^ + 0.1227*P*] where *P* = (*F*_o_^2^ + 2*F*_c_^2^)/3
2086 reflections	(Δ/σ)_max_ < 0.001
288 parameters	Δρ_max_ = 0.19 e Å^−^^3^
5 restraints	Δρ_min_ = −0.17 e Å^−^^3^

### Special details

**Table:**

Geometry. All e.s.d.'s (except the e.s.d. in the dihedral angle between two l.s. planes) are estimated using the full covariance matrix. The cell e.s.d.'s are taken into account individually in the estimation of e.s.d.'s in distances, angles and torsion angles; correlations between e.s.d.'s in cell parameters are only used when they are defined by crystal symmetry. An approximate (isotropic) treatment of cell e.s.d.'s is used for estimating e.s.d.'s involving l.s. planes.
Refinement. Refinement of *F*^2^ against ALL reflections. The weighted *R*-factor *wR* and goodness of fit *S* are based on *F*^2^, conventional *R*-factors *R* are based on *F*, with *F* set to zero for negative *F*^2^. The threshold expression of *F*^2^ > σ(*F*^2^) is used only for calculating *R*-factors(gt) *etc*. and is not relevant to the choice of reflections for refinement. *R*-factors based on *F*^2^ are statistically about twice as large as those based on *F*, and *R*- factors based on ALL data will be even larger.

### Fractional atomic coordinates and isotropic or equivalent isotropic displacement parameters (Å^2^)

**Table:**

	*x*	*y*	*z*	*U*_iso_*/*U*_eq_	
C1	0.31252 (16)	0.2574 (8)	0.5321 (2)	0.0467 (10)	
H1	0.3358	0.1258	0.5156	0.056*	
C2	0.28978 (16)	0.4345 (8)	0.4846 (2)	0.0538 (11)	
H2	0.2975	0.4226	0.4347	0.065*	
C3	0.25529 (18)	0.6318 (8)	0.5097 (3)	0.0548 (11)	
H3	0.2410	0.7496	0.4758	0.066*	
C4	0.24142 (16)	0.6603 (7)	0.5822 (3)	0.0504 (11)	
H4	0.2180	0.7923	0.5981	0.061*	
C5	0.26436 (14)	0.4805 (7)	0.6308 (2)	0.0409 (9)	
C6	0.29941 (15)	0.2824 (7)	0.60581 (19)	0.0391 (9)	
C7	0.29322 (16)	0.2498 (7)	0.7275 (2)	0.0397 (9)	
C8	0.30248 (17)	0.1623 (8)	0.8034 (2)	0.0487 (10)	
H8A	0.3179	−0.0129	0.8023	0.058*	
H8B	0.2675	0.1516	0.8282	0.058*	
C9	0.34010 (15)	0.3376 (7)	0.8482 (2)	0.0443 (10)	
H9A	0.3235	0.5092	0.8537	0.053*	
H9B	0.3745	0.3594	0.8224	0.053*	
C10	0.35076 (16)	0.2195 (7)	0.9238 (2)	0.0440 (10)	
H10A	0.3159	0.1796	0.9466	0.053*	
H10B	0.3703	0.0555	0.9176	0.053*	
C11	0.38324 (16)	0.3936 (8)	0.9753 (2)	0.0445 (10)	
H11A	0.3640	0.5584	0.9818	0.053*	
H11B	0.4185	0.4316	0.9534	0.053*	
C12	0.39191 (15)	0.2656 (7)	1.0503 (2)	0.0478 (11)	
H12A	0.3571	0.1985	1.0675	0.057*	
H12B	0.4163	0.1173	1.0442	0.057*	
C13	0.41530 (16)	0.4448 (8)	1.1086 (2)	0.0479 (10)	
H13A	0.3891	0.5820	1.1197	0.057*	
H13B	0.4483	0.5271	1.0901	0.057*	
C14	0.42850 (16)	0.2939 (7)	1.1783 (2)	0.0475 (9)	
H14A	0.3978	0.1810	1.1899	0.057*	
H14B	0.4598	0.1819	1.1688	0.057*	
C15	0.44061 (15)	0.4573 (7)	1.2432 (2)	0.0415 (9)	
C16	0.47804 (15)	0.7535 (7)	1.3163 (2)	0.0424 (9)	
C17	0.44065 (15)	0.6095 (8)	1.3572 (2)	0.0442 (10)	
C18	0.43226 (17)	0.6605 (9)	1.4312 (2)	0.0579 (12)	
H18	0.4077	0.5627	1.4589	0.070*	
C19	0.46186 (18)	0.8618 (10)	1.4617 (3)	0.0627 (12)	
H19	0.4571	0.9006	1.5112	0.075*	
C20	0.4989 (2)	1.0102 (10)	1.4205 (3)	0.0607 (12)	
H20	0.5176	1.1473	1.4428	0.073*	
C21	0.50813 (17)	0.9566 (8)	1.3469 (2)	0.0528 (11)	
H21	0.5333	1.0519	1.3194	0.063*	
N1	0.31566 (11)	0.1448 (6)	0.66844 (17)	0.0403 (7)	
H1A	0.3371	0.0113	0.6685	0.048*	
N2	0.26120 (12)	0.4500 (6)	0.70671 (16)	0.0417 (8)	
H2A	0.2418	0.5447	0.7357	0.050*	
N3	0.41815 (12)	0.4278 (6)	1.30898 (16)	0.0429 (8)	
H3A	0.3935	0.3142	1.3201	0.051*	
N4	0.47645 (12)	0.6521 (6)	1.24534 (17)	0.0430 (8)	
H4A	0.4956	0.7069	1.2088	0.052*	
N5	0.32105 (13)	0.8845 (7)	1.2993 (2)	0.0466 (8)	
O1	0.32896 (15)	0.9063 (7)	1.23308 (19)	0.0857 (11)	
O2	0.34026 (11)	1.0441 (6)	1.34445 (16)	0.0608 (8)	
O1W	0.46016 (12)	0.2164 (7)	0.62756 (17)	0.0558 (8)	
O3	0.29200 (12)	0.6979 (5)	1.32219 (16)	0.0584 (8)	
Cl1	0.40293 (4)	−0.28231 (18)	0.68522 (7)	0.0628 (3)	
H1WA	0.4422 (19)	0.087 (7)	0.654 (3)	0.11 (2)*	
H1WB	0.4403 (18)	0.347 (6)	0.633 (3)	0.10 (2)*	

### Atomic displacement parameters (Å^2^)

**Table:**

	*U*^11^	*U*^22^	*U*^33^	*U*^12^	*U*^13^	*U*^23^
C1	0.039 (2)	0.050 (2)	0.051 (3)	−0.0075 (19)	0.001 (2)	−0.007 (2)
C2	0.052 (3)	0.058 (3)	0.051 (3)	−0.013 (2)	−0.005 (2)	0.001 (2)
C3	0.055 (3)	0.058 (3)	0.052 (3)	−0.014 (2)	−0.015 (2)	0.010 (2)
C4	0.041 (2)	0.039 (2)	0.072 (3)	−0.0037 (18)	−0.011 (2)	0.003 (2)
C5	0.038 (2)	0.042 (2)	0.042 (2)	−0.0061 (18)	−0.0078 (19)	−0.0017 (19)
C6	0.034 (2)	0.040 (2)	0.043 (2)	−0.0050 (17)	−0.0119 (18)	−0.0048 (18)
C7	0.036 (2)	0.040 (2)	0.043 (2)	−0.0067 (18)	−0.0029 (18)	0.0000 (18)
C8	0.047 (2)	0.049 (2)	0.050 (3)	−0.009 (2)	−0.0036 (19)	0.003 (2)
C9	0.044 (2)	0.040 (2)	0.049 (2)	−0.0033 (18)	−0.0060 (19)	0.0042 (19)
C10	0.043 (2)	0.041 (2)	0.048 (2)	−0.0021 (18)	−0.0019 (19)	0.0014 (19)
C11	0.039 (2)	0.050 (2)	0.045 (2)	0.0013 (18)	−0.0053 (18)	0.0036 (19)
C12	0.046 (3)	0.048 (3)	0.049 (3)	−0.0009 (19)	−0.001 (2)	0.004 (2)
C13	0.046 (2)	0.044 (2)	0.053 (2)	−0.0043 (19)	−0.006 (2)	0.004 (2)
C14	0.055 (2)	0.043 (2)	0.044 (2)	0.0017 (18)	−0.004 (2)	−0.001 (2)
C15	0.039 (2)	0.040 (2)	0.046 (2)	0.0059 (18)	−0.0013 (19)	0.004 (2)
C16	0.035 (2)	0.044 (2)	0.049 (2)	0.0078 (18)	−0.0055 (19)	−0.002 (2)
C17	0.037 (2)	0.049 (2)	0.046 (2)	0.0086 (19)	−0.002 (2)	−0.005 (2)
C18	0.049 (3)	0.075 (3)	0.050 (3)	0.008 (2)	0.002 (2)	−0.004 (2)
C19	0.056 (3)	0.076 (3)	0.055 (3)	0.010 (3)	−0.009 (2)	−0.022 (3)
C20	0.058 (3)	0.052 (3)	0.072 (3)	0.007 (2)	−0.025 (2)	−0.010 (2)
C21	0.042 (2)	0.050 (3)	0.066 (3)	0.005 (2)	−0.015 (2)	0.000 (2)
N1	0.0339 (16)	0.0401 (17)	0.047 (2)	0.0016 (14)	−0.0051 (16)	−0.0020 (16)
N2	0.0368 (17)	0.0398 (18)	0.049 (2)	0.0008 (15)	−0.0004 (15)	−0.0055 (15)
N3	0.0347 (17)	0.0455 (19)	0.048 (2)	0.0013 (15)	0.0025 (15)	0.0002 (17)
N4	0.0441 (19)	0.0424 (18)	0.043 (2)	0.0015 (15)	−0.0007 (16)	0.0003 (15)
N5	0.0362 (18)	0.042 (2)	0.062 (2)	0.0067 (16)	−0.0008 (18)	0.0029 (19)
O1	0.105 (3)	0.096 (3)	0.056 (2)	−0.042 (2)	0.014 (2)	−0.002 (2)
O2	0.0548 (18)	0.0584 (18)	0.069 (2)	−0.0157 (15)	0.0000 (16)	−0.0166 (17)
O1W	0.0483 (18)	0.058 (2)	0.061 (2)	−0.0063 (16)	0.0040 (15)	−0.0109 (17)
O3	0.0575 (18)	0.0548 (18)	0.0628 (19)	−0.0141 (15)	−0.0010 (16)	0.0030 (15)
Cl1	0.0477 (6)	0.0446 (5)	0.0960 (9)	0.0035 (5)	−0.0071 (6)	0.0021 (6)

### Geometric parameters (Å, °)

**Table:**

C1—C2	1.369 (5)	C13—C14	1.519 (5)
C1—C6	1.385 (5)	C13—H13A	0.9700
C1—H1	0.9300	C13—H13B	0.9700
C2—C3	1.391 (6)	C14—C15	1.477 (5)
C2—H2	0.9300	C14—H14A	0.9700
C3—C4	1.372 (6)	C14—H14B	0.9700
C3—H3	0.9300	C15—N3	1.326 (5)
C4—C5	1.393 (5)	C15—N4	1.326 (5)
C4—H4	0.9300	C16—C21	1.387 (5)
C5—N2	1.393 (4)	C16—C17	1.389 (5)
C5—C6	1.402 (5)	C16—N4	1.393 (5)
C6—N1	1.397 (4)	C17—C18	1.388 (5)
C7—N1	1.320 (5)	C17—N3	1.390 (5)
C7—N2	1.341 (4)	C18—C19	1.374 (6)
C7—C8	1.471 (5)	C18—H18	0.9300
C8—C9	1.520 (5)	C19—C20	1.398 (7)
C8—H8A	0.9700	C19—H19	0.9300
C8—H8B	0.9700	C20—C21	1.387 (6)
C9—C10	1.527 (5)	C20—H20	0.9300
C9—H9A	0.9700	C21—H21	0.9300
C9—H9B	0.9700	N1—H1A	0.8600
C10—C11	1.516 (5)	N2—H2A	0.8600
C10—H10A	0.9700	N3—H3A	0.8600
C10—H10B	0.9700	N4—H4A	0.8600
C11—C12	1.528 (5)	N5—O1	1.227 (4)
C11—H11A	0.9700	N5—O2	1.248 (4)
C11—H11B	0.9700	N5—O3	1.259 (4)
C12—C13	1.514 (5)	O1W—H1WA	0.927 (19)
C12—H12A	0.9700	O1W—H1WB	0.83 (2)
C12—H12B	0.9700		
			
C2—C1—C6	117.2 (4)	H12A—C12—H12B	107.5
C2—C1—H1	121.4	C12—C13—C14	111.1 (3)
C6—C1—H1	121.4	C12—C13—H13A	109.4
C1—C2—C3	121.1 (4)	C14—C13—H13A	109.4
C1—C2—H2	119.4	C12—C13—H13B	109.4
C3—C2—H2	119.4	C14—C13—H13B	109.4
C4—C3—C2	122.9 (4)	H13A—C13—H13B	108.0
C4—C3—H3	118.6	C15—C14—C13	115.2 (3)
C2—C3—H3	118.6	C15—C14—H14A	108.5
C3—C4—C5	116.3 (4)	C13—C14—H14A	108.5
C3—C4—H4	121.9	C15—C14—H14B	108.5
C5—C4—H4	121.9	C13—C14—H14B	108.5
N2—C5—C4	133.0 (4)	H14A—C14—H14B	107.5
N2—C5—C6	106.0 (3)	N3—C15—N4	109.5 (3)
C4—C5—C6	121.0 (4)	N3—C15—C14	125.2 (4)
C1—C6—N1	132.7 (4)	N4—C15—C14	125.4 (4)
C1—C6—C5	121.5 (4)	C21—C16—C17	122.0 (4)
N1—C6—C5	105.7 (3)	C21—C16—N4	131.6 (4)
N1—C7—N2	108.8 (3)	C17—C16—N4	106.4 (3)
N1—C7—C8	125.3 (4)	C18—C17—C16	121.2 (4)
N2—C7—C8	125.9 (4)	C18—C17—N3	132.8 (4)
C7—C8—C9	114.7 (3)	C16—C17—N3	106.0 (3)
C7—C8—H8A	108.6	C19—C18—C17	117.1 (4)
C9—C8—H8A	108.6	C19—C18—H18	121.5
C7—C8—H8B	108.6	C17—C18—H18	121.5
C9—C8—H8B	108.6	C18—C19—C20	121.9 (4)
H8A—C8—H8B	107.6	C18—C19—H19	119.0
C8—C9—C10	110.8 (3)	C20—C19—H19	119.0
C8—C9—H9A	109.5	C21—C20—C19	121.2 (4)
C10—C9—H9A	109.5	C21—C20—H20	119.4
C8—C9—H9B	109.5	C19—C20—H20	119.4
C10—C9—H9B	109.5	C20—C21—C16	116.6 (4)
H9A—C9—H9B	108.1	C20—C21—H21	121.7
C11—C10—C9	114.6 (3)	C16—C21—H21	121.7
C11—C10—H10A	108.6	C7—N1—C6	110.0 (3)
C9—C10—H10A	108.6	C7—N1—H1A	125.0
C11—C10—H10B	108.6	C6—N1—H1A	125.0
C9—C10—H10B	108.6	C7—N2—C5	109.4 (3)
H10A—C10—H10B	107.6	C7—N2—H2A	125.3
C10—C11—C12	112.0 (3)	C5—N2—H2A	125.3
C10—C11—H11A	109.2	C15—N3—C17	109.3 (3)
C12—C11—H11A	109.2	C15—N3—H3A	125.3
C10—C11—H11B	109.2	C17—N3—H3A	125.3
C12—C11—H11B	109.2	C15—N4—C16	108.9 (3)
H11A—C11—H11B	107.9	C15—N4—H4A	125.6
C13—C12—C11	114.9 (3)	C16—N4—H4A	125.6
C13—C12—H12A	108.5	O1—N5—O2	121.9 (4)
C11—C12—H12A	108.5	O1—N5—O3	118.9 (4)
C13—C12—H12B	108.5	O2—N5—O3	119.2 (4)
C11—C12—H12B	108.5	H1WA—O1W—H1WB	104 (4)
			
C6—C1—C2—C3	0.4 (5)	N4—C16—C17—N3	−0.1 (4)
C1—C2—C3—C4	−0.8 (6)	C16—C17—C18—C19	1.0 (6)
C2—C3—C4—C5	0.6 (6)	N3—C17—C18—C19	−179.2 (4)
C3—C4—C5—N2	178.3 (4)	C17—C18—C19—C20	0.1 (6)
C3—C4—C5—C6	−0.2 (5)	C18—C19—C20—C21	−1.3 (7)
C2—C1—C6—N1	−178.1 (4)	C19—C20—C21—C16	1.4 (6)
C2—C1—C6—C5	0.0 (5)	C17—C16—C21—C20	−0.3 (5)
N2—C5—C6—C1	−179.0 (3)	N4—C16—C21—C20	178.8 (4)
C4—C5—C6—C1	−0.1 (5)	N2—C7—N1—C6	1.5 (4)
N2—C5—C6—N1	−0.4 (4)	C8—C7—N1—C6	−177.9 (3)
C4—C5—C6—N1	178.5 (3)	C1—C6—N1—C7	177.7 (4)
N1—C7—C8—C9	104.1 (4)	C5—C6—N1—C7	−0.7 (4)
N2—C7—C8—C9	−75.1 (5)	N1—C7—N2—C5	−1.8 (4)
C7—C8—C9—C10	−175.5 (3)	C8—C7—N2—C5	177.6 (3)
C8—C9—C10—C11	−174.1 (3)	C4—C5—N2—C7	−177.4 (4)
C9—C10—C11—C12	179.3 (3)	C6—C5—N2—C7	1.3 (4)
C10—C11—C12—C13	−169.9 (3)	N4—C15—N3—C17	−0.8 (4)
C11—C12—C13—C14	−173.5 (3)	C14—C15—N3—C17	177.8 (3)
C12—C13—C14—C15	−167.8 (3)	C18—C17—N3—C15	−179.2 (4)
C13—C14—C15—N3	130.3 (4)	C16—C17—N3—C15	0.5 (4)
C13—C14—C15—N4	−51.3 (5)	N3—C15—N4—C16	0.7 (4)
C21—C16—C17—C18	−0.9 (6)	C14—C15—N4—C16	−177.9 (3)
N4—C16—C17—C18	179.7 (3)	C21—C16—N4—C15	−179.7 (4)
C21—C16—C17—N3	179.3 (3)	C17—C16—N4—C15	−0.4 (4)

### Hydrogen-bond geometry (Å, °)

**Table:**

*D*—H···*A*	*D*—H	H···*A*	*D*···*A*	*D*—H···*A*
N1—H1A···Cl1	0.86	2.22	3.066 (3)	168
N2—H2A···O3^i^	0.86	1.94	2.778 (4)	164
N2—H2A···O1^i^	0.86	2.53	3.243 (4)	141
N2—H2A···N5^i^	0.86	2.59	3.436 (5)	168
N3—H3A···O2^ii^	0.86	1.95	2.807 (4)	177
N4—H4A···O1W^iii^	0.86	1.87	2.730 (4)	173
O1W—H1WA···Cl1	0.93 (2)	2.19 (2)	3.088 (4)	163 (4)
O1W—H1WB···Cl1^iv^	0.83 (2)	2.31 (3)	3.099 (3)	159 (5)
C10—H10A···Cg(3)^v^	0.97	3.36	4.148 (4)	140
C11—H11B···Cg(4)^vi^	0.97	3.21	4.047 (4)	146

Symmetry codes: (i) −*x*+1/2, *y*, *z*−1/2; (ii) *x*, *y*−1, *z*; (iii) −*x*+1, −*y*+1, *z*+1/2; (iv) *x*, *y*+1, *z*; (v) −*x*+1/2, *y*, *z*+1/2; (vi) −*x*, −*y*+1, *z*−1/2.
